# A minimal length rigid helical peptide motif allows rational design of modular surfactants

**DOI:** 10.1038/ncomms14018

**Published:** 2017-01-13

**Authors:** Sudipta Mondal, Maxim Varenik, Daniel Nir Bloch, Yoav Atsmon-Raz, Guy Jacoby, Lihi Adler-Abramovich, Linda J.W. Shimon, Roy Beck, Yifat Miller, Oren Regev, Ehud Gazit

**Affiliations:** 1 Department of Molecular Microbiology and Biotechnology, George S. Wise Faculty of Life Sciences, Tel Aviv University, Tel Aviv 69978, Israel; 2 Department of Chemical Engineering, Ben-Gurion University of the Negev, Be'er Sheva 84105, Israel; 3 Department of Chemistry, Ben-Gurion University of the Negev, Be'er Sheva 84105, Israel; 4 The Raymond and Beverly Sackler School of Physics and Astronomy, Tel Aviv University, Tel Aviv 69978, Israel; 5 Department of Oral Biology, The Goldschleger School of Dental Medicine, Tel Aviv University, Tel Aviv 69978, Israel; 6 Department of Chemical Research Support, Weizmann Institute of Science, Rehovot 76100, Israel; 7 Ilse Katz Institute for Nanoscale Science and Technology, Ben-Gurion University of the Negev, Be'er Sheva 84105, Israel; 8 Department of Materials Science and Engineering, Iby and Aladar Fleischman Faculty of Engineering, Tel Aviv University, Tel Aviv 69978, Israel

## Abstract

Extensive work has been invested in the design of bio-inspired peptide emulsifiers. Yet, none of the formulated surfactants were based on the utilization of the robust conformation and self-assembly tendencies presented by the hydrophobins, which exhibited highest surface activity among all known proteins. Here we show that a minimalist design scheme could be employed to fabricate rigid helical peptides to mimic the rigid conformation and the helical amphipathic organization. These designer building blocks, containing natural non-coded α-aminoisobutyric acid (Aib), form superhelical assemblies as confirmed by crystallography and microscopy. The peptide sequence is amenable to structural modularity and provides the highest stable emulsions reported so far for peptide and protein emulsifiers. Moreover, we establish the ability of short peptides to perform the dual functions of emulsifiers and thickeners, a feature that typically requires synergistic effects of surfactants and polysaccharides. This work provides a different paradigm for the molecular engineering of bioemulsifiers.

## Abstract (web summary)

Emulsifiers are used in the pharmaceutical, food, cosmetic, and biomedical industry. Here the authors fabricate rigid helical peptides that can perform as emulsifiers and thickeners, which typically requires synergistic effects of surfactants and polysaccharides.

Emulsifiers are a unique class of surfactant molecules that facilitate the dispersion of two immiscible liquids within a continuous liquid phase in the form of droplets, to generate a colloidal suspension^1^. These materials are central components in diverse applications in the pharmaceutical, food, cosmetic and biomedical industry^2^,^3^,^4^,^5^. The classical amphiphilic organic surfactants are most often used as components for emulsion preparation, but they exhibit various toxic and irritant properties^6^. Further, stability in emulsion formulation is accomplished by the addition of non-adsorbing polymers, such as different polysaccharides, including carboxymethyl cellulose and xanthan gum, which increases the viscosity of the bulk continuous phase to retard the coalescence of droplets and creaming—the major destabilizing processes^7^. However, a recent finding suggested that commonly used food grade surfactants such as Tween-80 and carboxymethyl cellulose are linked to the increase in inflammatory bowel disease observed since the mid-twentieth century and induced low-grade inflammation and obesity-metabolic syndrome in wild-type hosts^8^.

The biocompatibility and biodegradability of protein molecules make them one of the most attractive emulsifiers compared with the synthetic small molecule surfactants^9^,^10^. The common protein emulsifiers can be broadly classified into two distinct categories based on the nature of their interfacial adsorption. The majority of the proteins such as caseins, whey proteins and β-lactoglobulin undergo several degrees of denaturation or unfolding during and after adsorption, resulting in the exposure of alternative surface amino acid residues, leading to new protein–protein interactions in the adsorbed state^11^,^12^,^13^. Hydrophobins represent the second class of protein surfactants that maintained the native conformational state throughout the interfacial self-assembly processes^14^. They exhibit the highest surface activity among all known proteins and shows preferential adsorptions at the hydrophobic–hydrophilic interfaces in the presence of other proteins^15^. This distinctive characteristic of hydrophobins stems from their compact and robust tertiary structure ascribed to the presence of conserved cross-linked cysteine residues^16^.

Peptides possess all the natural properties of proteins, but at the same time have highly modular structural features and can be tailored to accommodate essential attributes required for specific applications^17^. Thus, peptide emulsifiers are highly anticipated for drug delivery and related applications. So far, the design of *de novo* peptide-based emulsifiers has focused on mimicking proteins with flexible backbone conformations. In such pioneering approach, Middelberg and colleagues^18^,^19^ developed a helical peptide surfactant comprising 21 amino acids that formed a switchable cohesive interfacial film assisted by metal ions and produced oil-in-water emulsions with stability against coalescence over 20 h on standing. In another study, Ulijn and colleagues^20^,^21^ demonstrated that several hydrogel-forming short peptide sequences could provide functional emulsion-stabilizing systems. Several other peptide sequences with conformational freedom were designed to evaluate the role of secondary structures and amphiphilic conformations in interfacial adsorption and emulsifying behaviours^22^,^23^,^24^,^25^.

In an alternative strategy, here we report the finding of a group of conformationally constrained peptide emulsifiers composed of seven amino acid residues that mimic the rigid conformational model of hydrophobins and afford oil-in-water emulsions with the highest long-term stability among all peptide-based emulsifiers reported so far. The designer peptides adopt rigid canonical helical conformation as confirmed by X-ray crystallography analysis and self-assemble to a nano-structured morphology in bulk aqueous solution as probe by cryo-transmission electron microscopy (cryo-TEM) and molecular simulation. In an unparalleled manifestation of the structure–function relationship, we observe that the peptide retains its excellent emulsification behaviour even after structural modifications at the core positions.

## Results

### Structure and self-assembly of designed helical peptide

**Figure 1:**
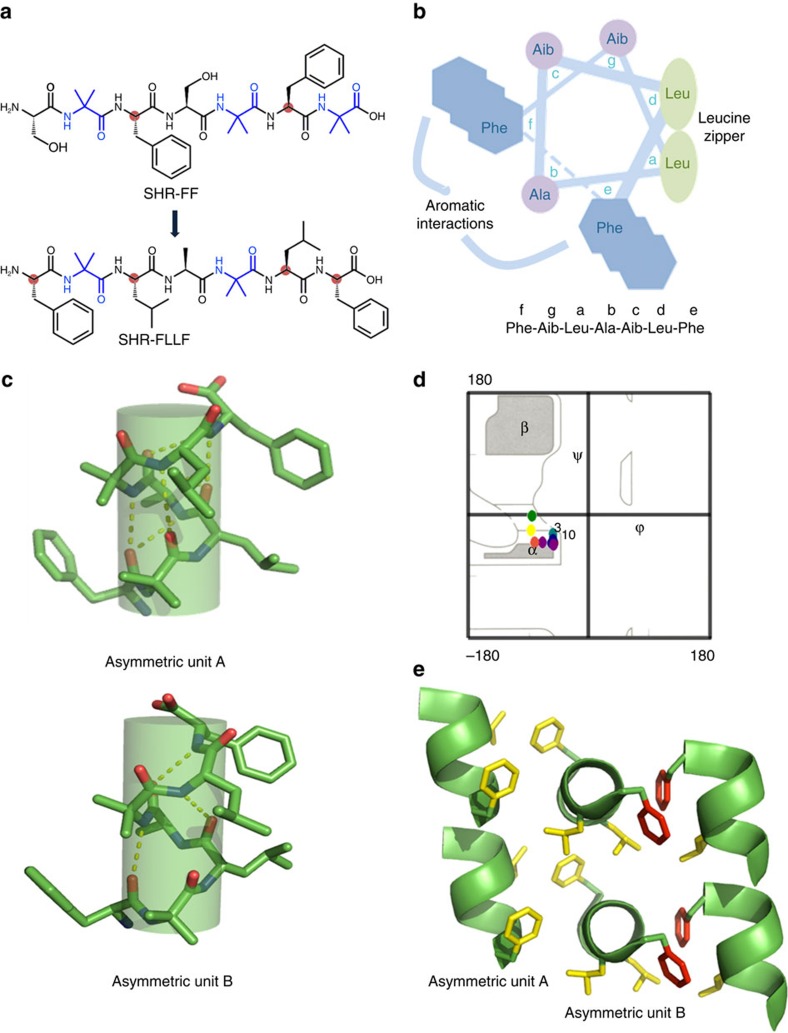
Design scheme and single crystal X-ray analysis of SHR-FLLF. (**a**) Sequence modification of SHR-FF to afford SHR-FLLF with higher helical propensity. Major residues involved in sequence modification are highlighted. (**b**) Helical wheel representation of designed SHR-FLLF heptad peptide showed the predicted relative position of the amino acids in the helix. The width of the line decreases from the C terminus to N terminus. The flexible terminal Phe residues at e,f can favourably form *π*-stacking with Phe of neighbouring helical modules. The leucine residues present at a,d positions may afford supramolecular zipper structure owing to favourable hydrophobic interactions. (**c**) Depiction of canonical helical conformation of the two asymmetric units of SHR-FLLF observed in crystal as cylindrical helices. (**d**) Torsion angles of the asymmetric units (coloured round symbols) superimposed over ideal Ramachandran plot. (**e**) Packing of adjacent head-to-tail helical columns of SHR-FLLF revealed perpendicular packing pattern and stabilized by hydrophobic and stacking interactions as shown by coloured amino acid side chains (only relevant amino acid side chains are displayed). The Phe and Leu residues in yellow represent residues that participate in ‘knob into hole' structures. The Phe residues in red represent *π*-stacking interactions.

The design of the peptides was based on our recent efforts to explore and understand the supramolecular helical assembly by minimal heptad peptide motifs. Recently, we reported the solution state morphology and solid-state structure of the peptide SHR-FF (H_2_N-Ser-Aib-Phe-Ser-Aib-Phe-Aib-OH; Fig. 1a), containing α-aminoisobutyric acid residues (Aib)^26^. We observed that all the amino acid residues, except phenylalanine (Phe), adopted dihedral angles in accordance with a Ramachandran plot of a 3_10_ helix^26^. The discrepancy in dihedral angles of Phe prompted us to refine the original design by modifying SHR-FF. This afforded the peptide SHR-FLLF, in which the phenylalanines at positions four and seven were replaced with helix-favouring leucine residues (Fig. 1a). The remarkable aggregation propensity of Phe towards supramolecular assembly^27^,^28^ was conserved by placing Phe at the terminus with minimal effect on helical folding (Fig. 1a). At the same time, serine was replaced with alanine, which has high helical propensity compared with all other amino acids. We envisioned that these structural modifications would constrain the SHR-FLLF sequence to adopt the classical helical folding and the conformational freedom of the terminal Phe residues would afford favourable *π*-stacking interactions that may generate self-assembled nanostructures with supramolecular organization of the helical peptides (Fig. 1b). In addition, the leucine residues at four and seven positions can form hydrophobic zipper-like region providing additional stability to the supramolecular organization of SHR-FLLF.

The preferred structural conformation of the SHR-FLLF was probed by X-ray crystallography analysis with crystals that were grown in phosphate buffer solution at physiological pH. The SHR-FLLF crystallized into two independent conformations as shown in Fig. 1c and the torsion angles of all residues coincided well with the Ramachandran plot of an ideal helical peptide, as hypothesized in the original design (Fig. 1d). The average *ϕ* and *ψ* angles of the asymmetric unit A were −63 and −39, respectively, and could be correlated with the right-handed α-helix found in proteins that have average dihedral angles of −64±7 and −41±7, respectively (Supplementary Table 1). The molecule was composed of three *i*, *i*+4 intra-helical hydrogen bonds, consistent with the maximum intramolecular NH····O=C interactions possible for a seven-residue helical sequence (Fig. 1c). In addition, all relevant amide nitrogens formed bifurcated *i*, *i*+3 hydrogen bonding with their respective carbonyl groups. The asymmetric unit B adopted a similar backbone conformation with average *ϕ* and *ψ* angles of −65 and −32, respectively, and with slight differences from the ideal α-helical conformation observed in molecule A (Fig. 1c). The molecule showed two intra-helical *i*, *i*+3 and one *i*, *i*+4 hydrogen-bonding motifs and thus assumed a conformation with mixed 3_10_/α-helical character.

The asymmetric units A and B formed continuous head-to-tail helical columns where the helical axis of column A was oriented perpendicularly to the helical axis of column B, and all the amide groups and the terminal amine moiety were located at the amino terminus of one helix, which formed hydrogen bonds with the carboxy-terminal carbonyl groups of the helical unit situated underneath (Fig. 1e and Supplementary Fig. 1). Helical layers of molecules B interacted with adjacent helical layers of A by different kinds of hydrophobic and *π*-stacking; in one facet, the complex adopted ‘knob-into-hole'-type arrangements involving Phe and leucine residues (marked by yellow colour, Fig. 1e) and the complementary interface was stabilized by efficient *π*-stacking mode involving Phe (Phe residues in red, Fig. 1e). To the best of our knowledge, this is the only example of a minimal heptapeptide with a free terminus composed singularly of natural amino acids adopting the canonical helical conformation in the solid state^29^. Such structural features with free amino and carboxyl groups are critical for harnessing the material properties of these building blocks, as will be discussed below.

**Figure 2:**
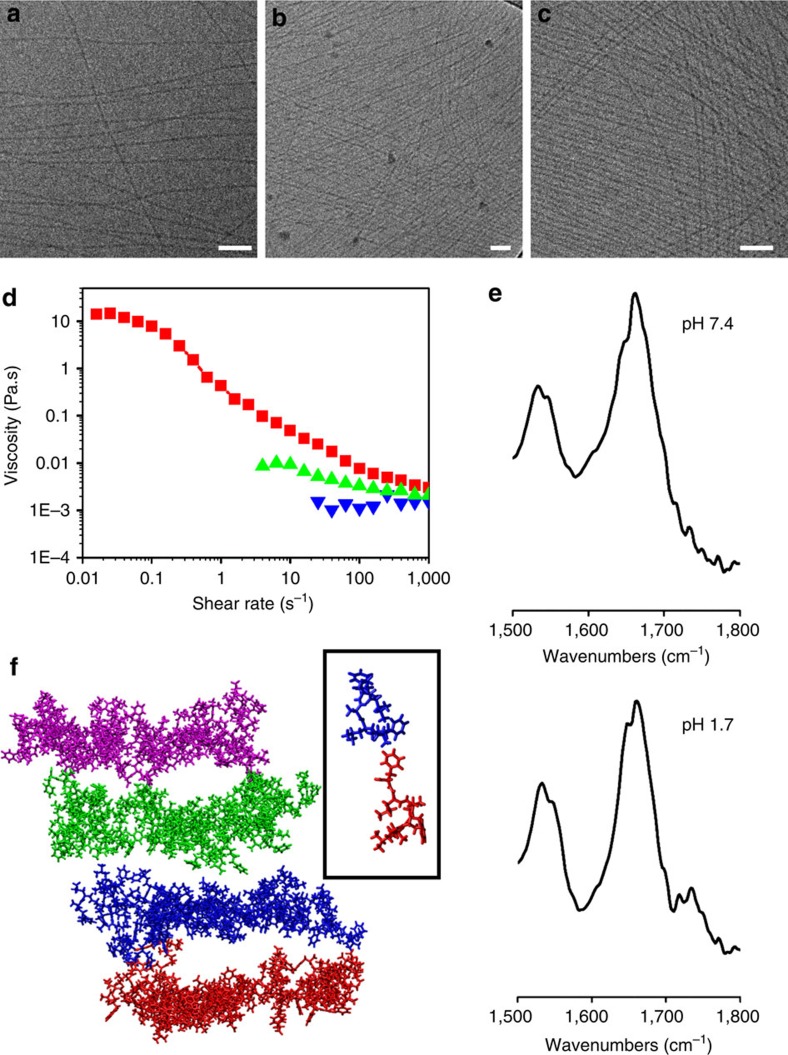
Validation of self-assembly and structural rigidity of SHR-FLLF. (**a**–**c**) Cryo-TEM micrograph of cylindrical micelles-like nanofibres formed at concentrations of (**a**) 5, (**b**) 10 and (**c**) 15 mg ml^−1^ in aqueous solution. Scale bars, 50 nm. (**d**) Viscosity versus shear rate profile of different concentration of peptides confirmed apparent viscoelastic behaviour on increasing peptide concentrations (from bottom to top; colour code: 5 mg ml^−1^, blue; 10 mg ml^−1^, green; 15 mg ml^−1^, red). (**e**) FTIR peaks of SHR-FLLF at neutral (pH 7.4) (top) and acidic pH (bottom). (**f**) A view of the fibril-like structure from molecular dynamics simulation. The fibril-like structure consisted of four layers. Each layer is presented in a different colour. Inset: a zoom of *π*–*π* interactions viewed between the blue layer and the red layer.

The supramolecular assembly of SHR-FLLF was explored by using cryo-TEM, as it provides near-native structural features present in solution. The peptide exhibited limited solubility and precipitated in phosphate buffer at pH 7.4, conditions similar to the crystallization. The presence of the ionic free terminus allowed the modulation of peptide solubility by altering the solution pH^30^. SHR-FLLF was dissolved in concentrations ranging from 5 to 20 mg ml^−1^ in water at pH below 2, to obtain transparent solutions and no precipitation was observed after long-term storage at room temperature. The zeta potential of the peptide solution showed considerable positive value at this pH, confirming the charged state of the peptide (Supplementary Table 2). The cryo-TEM revealed that the peptide self-assembled into uniform high aspect ratio nanofibres of 5–6 nm diameter that extended for several micrometres (aspect ratio, *L*/*D*>500). The nanofibre morphology was independent of peptide concentration and showed identical structure in all concentration regimes (Fig. 2a–c and Supplementary Fig. 2a). To compare, electron microscopy of the precipitate at pH 7.4 was performed and formation of irregular structures with a wide size distribution was observed (Supplementary Fig. 2b). Despite the identical bulk solution morphology, the viscoelastic nature of the peptide solution was changed substantially on increasing the peptide concentration (Fig. 2d). At 5 mg ml^−1^, SHR-FLLF showed Newtonian fluid behaviour and was independent of the applied shear. At higher concentrations, shear thinning occurred as evidenced by a decrease in viscosity with increased shear rate. Many amphiphilic molecules with cylindrical micelles assembly have revealed concentration-dependent shear thinning^31^ and, in an analogy, it can be assumed that at 5 mg ml^−1^ the nanofibres in the system are separated by long distance and have negligible influence on viscosity, whereas at relatively high concentration they become entangled with each other, forming transient networks with enhanced viscoelastic properties.

The conformational states of the SHR-FLLF at pH 7.4 and acidic pH were compared using Fourier transform infrared (FTIR) spectroscopy. The amide I and amide II bands at 1,661 and 1,534 cm^−1^, respectively, confirmed the presence of helical conformation observed in the crystal structure^32^ (Fig. 2e). Similar amide bands were a spectral feature at pH 2 and established that the conformation of SHR-FLLF did not deviate considerably in acidic pH (Fig. 2e). Additional support for helical conformations was obtained by measuring FTIR spectra in D_2_O solution. As shown in Supplementary Fig. 3a, the amide I bands at 1,642 and 1,649 cm^−1^ indicated that peptide adopted both 3_10_ and α-helical conformations in solution, which is in agreement with the secondary structure observed in X-ray crystallographic analysis^33^. This observation can be ascribed to the rigid conformationally locked backbone of the designed peptide. Further insights about conformational rigidity and self-assembly of SHR-FLLF were ascertained by molecular dynamic simulation. It has been observed that SHR-FLLF preserved its helical conformation in a simulated model. To understand the supramolecular interactions in nanofibres, a fibril-like structural model was constructed by forming four layers, in which each layer consisted of 4 peptides by 6 peptides (that is, a total of 24 peptides), to form a diameter of ∼5–6 nm as observed by the cryo-TEM measurements (Fig. 2f and Supplementary Note 1). The four peptides in each layer formed *π*–*π* interactions between the two Phe residues. The six peptides in each layer formed hydrophobic interactions between the Aib residue and the Ala residue. After simulations were performed, the diameter values of the fibril-like structure were 6.2 nm by 5.1 nm, indicating that the diameter values were conserved within the time scale of the simulation. In addition to intra-fibril interactions, the modelled fibril also formed hydrophobic interactions with neighbouring fibres and may account for a viscoelastic network at higher concentration (Supplementary Fig. 4). The wide-angle X-ray scattering (WAXS) spectra of the nanofibre solution were recorded at two different concentrations, to understand the absolute molecular arrangement of peptide in nanofibresers (Supplementary Fig. 5). However, under the current experimental conditions, the resolution of the spectra impede further specific conclusion. The cumulative single crystal and FTIR studies confirmed that SHR-FLLF adopted a rigid rod-like conformation in which the helical segment constituted a cylindrical hydrophobic environment, with the apex and base of the cylinder being composed of hydrophilic terminal groups and thus resembling a bolaamphiphilic structure (Fig. 1c). The arrangement of such cylindrical helical motifs in self-assembled nanofibres is better illustrated by molecular dynamic simulation presented in Fig. 2f. The designer sequence affirmed a different class of self-assembling bolaamphiphilic helical peptide composed of natural amino acids and provides a significant structural extension to the charged surfactant-like peptides designed by the research group of Zhang and colleagues^34^,^35^,^36^,^37^ and peptide amphiphiles designed by the research group of Stupp and colleagues^38^,^39^,^40^,^41^ in which the peptides adopt β-sheet conformation and self-assemble into a lipid-like bilayer to shield the hydrophobic segments from the aqueous environment.

### Features of emulsions stabilized by the peptide amphiphile

**Figure 3:**
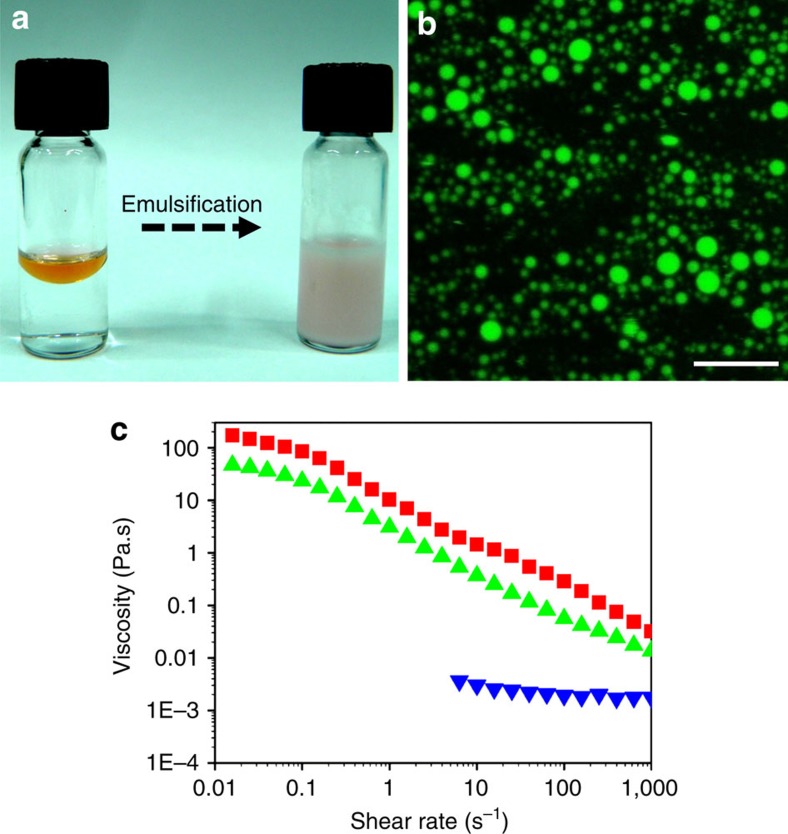
Characterization of SHR-FLLF stabilized emulsion. (**a**) Photographic image of silicone oil (20%) and water (80%) emulsions before and after emulsifications. Silicone oil was stained with Sudan III dye. (**b**) Fluorescence micrograph demonstrates the formation of oil-in-water emulsion. Oil droplets fluoresce because of entrapped Nile red dye. Scale bar, 10 μm. (**c**) Apparent viscosity versus shear rate profile of emulsions prepared with 5, 10 and 15 mg ml^−1^ of SHR-FLLF, respectively (from bottom to top; colour code: 5 mg ml^−1^, blue; 10 mg ml^−1^, green; 15 mg ml^−1^, red) showed thickening or stabilizing proficiency of peptide emulsifiers.

The amphiphilic nature of SHR-FLLF and the similarities with hydrophobins in terms of structural rigidity and nanofibrillar assembly^42^ inspired us to study the characteristic of this peptide as an emulsifier, to develop peptide-based biosurfactants with a minimal helical peptide sequence. To understand the critical aggregation concentration (CAC) of SHR-FLLF in aqueous solution, above which surfactants exhibit high emulsification activity, a pyrene fluorescence assay was adopted and a CAC value of 2.8 mg ml^−1^ was measured (Supplementary Fig. 6). An emulsion was then prepared by dissolving SHR-FLLF in water at pH below 2 at a concentration of 5 mg ml^−1^, a value higher than the CAC, and the aqueous peptide solution was subsequently emulsified with silicone oil with an oil volume fraction of 0.2. Silicone oil was preferable for this procedure due to its extensive applications in cosmetics and pharmaceuticals, and because difficulties had arisen in emulsifications from the unique chemical structure of the silicone polymer^43^. As shown in Fig. 3a, the SHR-FLLF efficiently emulsified the silicon oil–water mixture. Confocal fluorescence microscopy was employed to ascertain the nature of the emulsion by labelling the silicone oil with Nile Red dye and the presence of brightly fluorescent oil droplets confirmed the formation of the oil-in-water emulsion (Fig. 3b). The emulsions were further characterized by measuring the hydrodynamic diameters and surface charges of oil droplets (Table 1 and Supplementary Fig. 7). To compare the emulsification activity of SHR-FLLF with the most widely used commercial emulsifiers such as SDS and Tween 20, emulsions were prepared under similar conditions and the physical parameters of such emulsions are listed in Table 1. Phase separation and creaming could be observed for oil-in-water systems prepared with Tween 20 after 2 days; emulsions prepared with SHR-FLLF and SDS exhibited identical stability over time and were stable for weeks (Supplementary Fig. 8). This similar behaviour is also supported by the comparable mean particle sizes of SHR-FLLF and SDS emulsions, whereas oil droplets sizes of Tween 20-stabilized emulsion was much higher (Table 1). Very high zeta potential (Table 1) and comparable physical characteristic with SDS confirmed that the SHR-FLLF was highly surface active and formed strong interfacial networks. To understand the conformational preference of SHR-FLLF at oil–water interface, emulsions were prepared in D_2_O and FTIR spectra were measured in solution state. The bands at 1,642 and 1,649 cm^−1^ demonstrated excellent correlation with the SHR-FLLF conformation observed in D_2_O (Supplementary Fig. 3b). Furthermore, drying of the emulsion (prepared in H_2_O) over KBr crystal revealed amide I band at 1,662 cm^−1^, which is consistent with helical conformation of the designed peptide (Supplementary Fig. 3c). These studies confirmed that the SHR-FLLF conserved the helical conformation at the oil–water interface and self-assembled to supramolecular helical arrangement to stabilize the emulsion.

The preservation of viscoelastic properties of native SHR-FLLF at relatively high concentration would render the emulsion viscoelastic and may impart additional stability against coalescence and creaming. Based on this supposition, SHR-FLLF stabilized emulsions were prepared by dissolving the peptide at 10 and 15 mg ml^−1^ concentrations, and rheological properties and long-term stability were studied. Zeta potential and droplets sizes of the prepared emulsions had similar characteristics as the emulsion prepared with 5 mg ml^−1^ of SHR-FLLF (Table 1 and Supplementary Fig. 7). However, the formed emulsions exhibited minimum stability at two months and several batches of preparations showed no signs of phase separation even after 8 months of storage (Supplementary Fig. 9). All the stability analysis was performed at 25 °C, to simulate a real-life environmental condition. To the best our knowledge, the long-term emulsion stability presented by SHR-FLLF is the highest among the peptide-based emulsifiers described in the literature^18^,^20^,^21^,^22^,^23^. Rheology measurements were performed to investigate the viscoelastic nature of the emulsions. As shown in Fig. 3c, at 5 mg ml^−1^ of SHR-FLLF, emulsion showed Newtonian flow behaviour as expected for an emulsion with low oil content. However, viscoelastic properties of the emulsion increased dramatically at 10 and 15 mg ml^−1^ of SHR-FLLF and exhibited shear thinning behaviour. The measured viscosity was much higher than that of the corresponding peptide solutions and indicated that the peptide formed an assembled structure in continuous phase and afforded transient network structures that reduced the mobility of the oil droplets, which prohibited coalescence^44^, and consequently produced emulsion with exceptional stability. Rod-like polysaccharides and protein fibrils are known to impart additional stability to the emulsion compared with random coil structures owing to the higher excluded volume^45^. The highly stable emulsion with SHR-FLLF suggested that the rigid backbone may play a crucial role in self-assembly and viscoelastic emulsion formation.

### Versatility of rigid minimal helical peptide as emulsifiers

**Figure 4:**
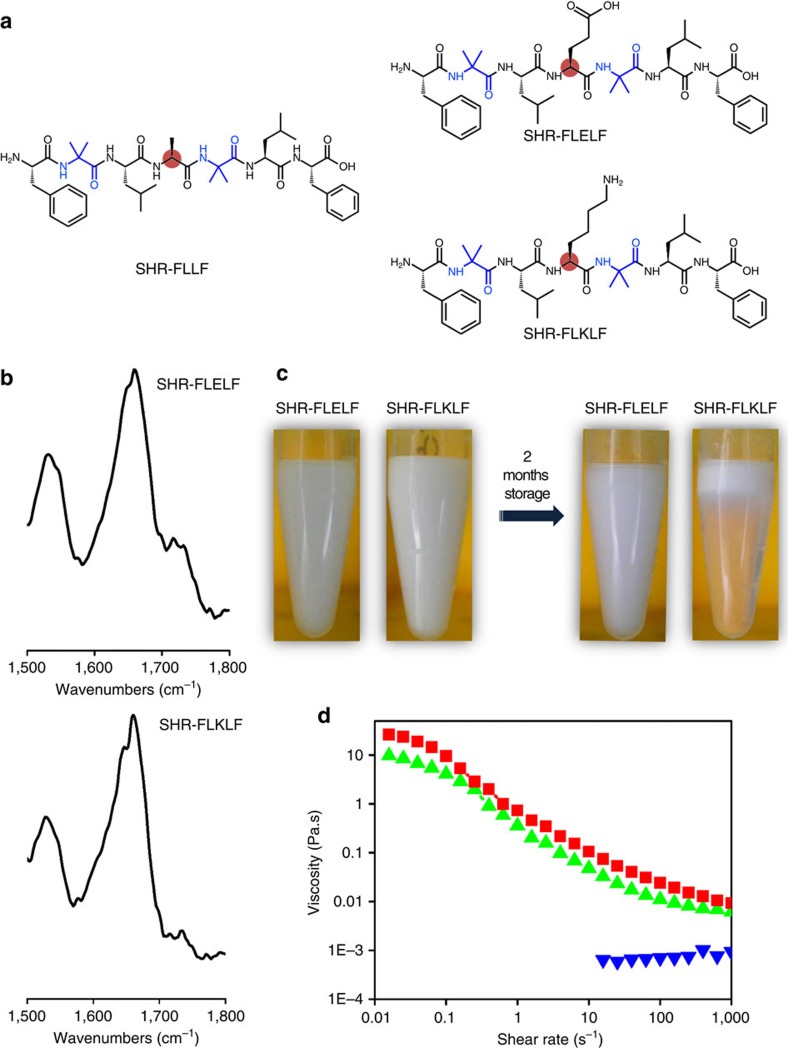
Demonstration of structural modularity of conformationally constrained helical peptide emulsifiers. (**a**) Structural modification of SHR-FLLF to afford SHR-FLELF and SHR-FLELF. Major residues involved in sequence modification are highlighted. (**b**) Truncated FTIR spectra of SHR-FLELF (top) and SHR-FLKLF (bottom). (**c**) Photographic images depicting the long-term stability of emulsions prepared with SHR-FLELF (7.5 mg ml^−1^) and SHR-FLELF (7.5 mg ml^−1^). (**d**) Profiles of apparent viscosity versus shear rate of emulsions prepared with 5, 7.5 and 10 mg ml^−1^ of SHR-FLELF, respectively (from bottom to top; colour code: 5 mg ml^−1^, blue; 7.5 mg ml^−1^, green; 10 mg ml^−1^, red) established general thickening or stabilizing proficiency of this class of peptide emulsifiers.

To demonstrate the structural versatility of this minimal conformationally constrained heptapeptide as an emulsifier and comprehend the relationship between the structure–assembly–function, we designed two additional minimal peptide sequences SHR-FLELF and SHR-FLKLF by replacing alanine with helix-favouring amino acids, glutamic acid and lysine (Fig. 4a). Such structural modification is expected to disrupt the bolaamphiphilic feature of SHR-FLLF and the retention of the native functionality will prove the excellent robustness of the system towards structural alteration. The secondary structural conformations of the peptides were probed by FTIR spectroscopy, both in the solution and after drying the samples over IR card. The presence of amide I peaks at 1,658 and 1,660 cm^−1^ for SHR-FLELF and SHR-FLKLF, respectively, in dry samples (prepared in H_2_O) indicated the expected helical conformation of the peptides (Fig. 4b). Furthermore, the peptide was dissolved in D_2_O and FTIR spectra were recorded in solution state, as it provides the information about the dynamic conformation. The spectra of SHR-FLELF exhibited two bands at 1,644 and 1,651 cm^−1^, confirming the presence of 3_10_ and α-helical components in the solution state (Supplementary Fig. 10a). Similar spectral features with amide I bands at 1,644 and 1,650 cm^−1^ of SHR-FLKLF indicated that the backbone conformation of the designed peptides are highly robust and have the potential to accommodate a variety of modification (Supplementary Fig. 10d). The pyrene fluorescence assay revealed the CAC of SHR-FLELF to be 1.5 mg ml^−1^ (Supplementary Fig. 11a,b). However, no significant change in the pyrene fluorescence peak intensity was evident in the presence of 10 mg ml^−1^ of SHR-FLKLF (Supplementary Fig. 11c), a result that was consistent with the very high solubility of the peptide (<100 mg ml^−1^) as compared with SHR-FLFLF (∼15 mg ml^−1^) around pH 2. This study confirmed that SHR-FLELF self-assembled into a micelle-like structure, whereas SHR-FLKLF persisted in the non-aggregated state in solution in the concentration range of 10 mg ml^−1^. The emulsification activities of SHR-FLELF and SHR-FLKLF were investigated under similar conditions as used with SHR-FLLF. SHR-FLKLF stabilized emulsions revealed a fast phase separation due to coalescence, although they also showed comparable droplet sizes and zeta potential with SHR-FLELF (Fig. 4c, Table 1 and Supplementary Figs 12 and 13). The backbone conformation of the peptides at oil–water interface was assessed by FTIR spectroscopy. The SHR-FLELF emulsions prepared in D_2_O revealed amide I bands at 1,645 and 1,654 cm^−1^, indicating the presence of helical conformation at the interface. The FTIR signals of SHR-FLKLF show a single broad peak at 1,644 cm^−1^ and can be attributed to the 3_10_ helical conformation (Supplementary Fig. 10d,e). The drying of the emulsions prepared with SHR-FLELF and SHR-FLKLF over KBr crystal displayed amide I peaks at 1,663 and 1,661 cm^−1^, respectively, indicating that the native helical conformation of the peptides was maintained under this conditions (Supplementary Fig. 10c,f). This outcome demonstrated that the designed helical peptides have excellent interfacial adsorption characteristics but the integrity of the interfacial peptide network was much more robust for the SHR-FLELF, owing to its high aggregation propensity in solution. The concentration-dependent viscoelastic nature of the SHR-FLELF was studied further, to gain insights about the effect of the self-assembly of peptide with a robust backbone conformation with respect to emulsion stability (Fig. 4d). At 5 mg ml^−1^, the viscosity of SHR-FLELF-stabilized emulsion was low and had a fluid-like flow. The viscosity of the emulsion was elevated sharply at a concentration of 7.5 mg ml^−1^ with a shear thinning effect. This emulsion was stable for an examination period of 2 months without any indication of phase separation or creaming (Fig. 4c). Further increases in concentration (10 mg ml^−1^) had a minimal effect on emulsion viscosity and gave a modest rise that could be accounted for by a higher peptide concentration in continuous phase. It is important to mention that high concentrations of most protein emulsifiers have detrimental effects on emulsion stability, and fast phase separation and creaming are common phenomena due to depletion flocculation^46^.

## Discussion

A direct correlation between the self-assembly features, helical folding pattern and emulsion stability of all the designed peptide sequences pertaining to all the tested concentrations was presented in Table 2. The combinations of single crystal analysis, molecular dynamic simulation and FTIR spectroscopy indicated that the peptides adopted robust helical backbone conformation. However, as probed by the electron microscopy and pyrene fluorescence assays, only the peptides SHR-FLLF and SHR-FLELF self-assembled to supramolecular helical arrangement, whereas SHR-FLKLF exists mainly in non-aggregated states. Highly stable emulsions formed by the former two sequences can be directly correlated with their high self-assembly propensity in solution. The robust conformation of these peptides may permit favourable interactions between the absorbed interfacial peptide layers and the self-assembled structure in bulk aqueous phase, and these are manifested by the increase in viscoelastic nature of the emulsions at relatively high peptide concentration.

In conclusion, a group of peptide amphiphiles with a rigid backbone conformation were designed and self-assembled into nanoscale morphology in aqueous solution. The soluble monodisperse cylindrical micelle structures will allow molecular engineering of supramolecular helical peptide assemblies based on minimal helical peptide sequence. The designer amphiphilic building blocks demonstrated excellent surface activity toward oil-in-water emulsion formation and maintained their propensity towards self-aggregation in emulsified continuous phase, revealing an extraordinarily stable emulsion with a single component acting as both emulsifier and stabilizer, a feature never realized before in peptide emulsifiers. The engineered building blocks showed high modularity and sequence–structure–function relationship. The versatility of such amino acid toolkits point to the incorporation of different features such as the elicitation of a response towards external stimuli and switchable surfactant behaviours. These materials may provide another paradigm for biosurfactants in applications related to drug delivery and the food and cosmetics industries.

## Methods

### General sample preparation

All the peptides were purchased from Peptron, Inc. (South Korea). SHR-FLLF was suspended in water at desired concentration and 1 N HCl was added to dissolve the peptide. Final pH was maintained in the range of 1.4–1.8. To prepare SHR-FLLF samples in buffer (pH 7.4), the peptide was dissolved in ethanol in 100 mg ml^−1^ concentration and diluted to 5 mg ml^−1^ with phosphate buffer (10 mM). SHR-FLELF and SHR-FLKLF were dissolved in water at preferred concentrations and 1 N HCl was added to adjust the pH in the range of 1.4–1.8. All the peptides formed transparent solutions under the conditions described above. Control experiments with SDS and Tween 20 were performed by dissolving the corresponding surfactants in water at the desired concentrations (SDS, 2.8 mg ml^−1^ and Tween 20, 1.2 mg ml^−1^) and final solution pH was maintained between 1.4 and 1.8.

### Cryo-transmission electron microscopy

Cryo-TEM samples were prepared using Leica EM GP cryo-preparation instrument operated at 100% relative humidity. Samples were prepared as described in general Methods section. Peptide solution (2.5 μl) was applied on a holey carbon TEM grid (Lacey substrate, 300 mesh, Ted Pella, Inc.) followed by blotting with a filter paper and plunging into liquid ethane. The vitrified samples were kept under liquid nitrogen and subsequently transferred to a FEI-Tecnai T12 TEM using a Gatan workstation and cryo-holder. The images were acquired at 98 K with an operating voltage of 120 kV in low electron dose mode. Images were recorded on a Gatan 794 charge-coupled device camera.

### Transmission electron microscopy

To prepare the samples for TEM imaging, peptides were dissolved in ethanol (100 mg ml^−1^) and diluted by the addition of phosphate buffer (pH 7.4) to reach a final concentration of 5 mg ml^−1^. A 7 μl aliquot of this solution was placed on a 400-mesh copper grid and excess fluids were removed by blotting with a filter paper after 2 min. The samples were negative stained with 2% uranyl acetate in water and excess fluids were removed from the grid after 2 min. TEM images were recorded using a JEM-1400 electron microscope (JEOL) operating at 80 kV.

### Bulk rheology

Rheological measurements of the emulsions were performed on a Discovery Hybrid Rheometer HR3 (TA Instruments, USA) equipped with cone and plate geometry (diameter, 40 mm; cone angle, 1.04°). All measurements were conducted in a steady-state shear sweep mode at a temperature of 25±0.1 °C after 24 h of samples preparation.

### Emulsion preparation and characterization

All the emulsions were prepared with silicone oil (Viscosity 10 cSt; Sigma-Aldrich) with oil volume fraction of 0.2. Peptide concentrations used for the emulsifications were as follows: 5, 10 and 15 mg ml^−1^ of SHR-FLLF; 5, 7.5 and 10 mg ml^−1^ of SHR-FLFLF; and 5 and 7.5 mg ml^−1^ of SHR-FLKLF, respectively. Peptide solutions with desired concentration were prepared by the methods described in general section followed by the addition of required amount of silicone oil. The combined solution was subjected to probe sonication (Syclon probe ultrasonicator: SKL-150-IIDN) for duration of two 5 s pulses at an output of 27%, whereas the delay between pulses is 9 s. For long-term stability assessment, the emulsions were stored at 25 °C and stability was monitored by visual observation and capturing photography images. The droplet size distribution of the emulsions was measured by diluting the samples by 20 times in corresponding continuous phase (Zetasizer Nano ZS, Malvern Instruments). The zeta potential measurement was performed by diluting the emulsions in double distilled water (Zetasizer Nano ZS, Malvern Instruments). Zeta potentials were also measured by diluting the emulsions in the corresponding acidic continuous phase and all the samples revealed zeta potential values above +100 mV. These high values are above the usual limit of the instrument and, therefore, zeta potential data were collected by diluting the emulsions in double distilled water. The SHR-FLELF has low isoelectronic point than the other peptides and consequently has lower zeta potential under this condition. For photography, Sudan III was dissolved in silicone oil before emulsion preparation.

### Confocal laser scanning microscopy

Confocal microscope images were recorded in a Zeiss LSM 510 META. The emulsion was prepared with silicone oil that was prestained with 1 mM Nile Red dye. Emulsion suspensions diluted tenfold with continuous phase. A drop of emulsion was deposited on glass slide and covered with glass coverslip. The imaging was performed with × 100 objective lens by exciting the sample at 477 nm and emission was collected in 540–657 channels.

### Critical aggregation concentration

The CAC value was determined by a modification of the methods described in ref. 47. Pyrene solution (5 mg) was dissolved in methanol and diluted 20 times in methanol to prepare the stock. Fixed concentration of pyrene stock was added to different peptide solutions with concentrations ranging from 0.1 to 10 mg ml^−1^ and the total volume was kept constant at 600 μl. Fluorescence spectra was recorded by exciting the samples at 334 nm and measuring the emission from 360 to 410 nm with the use of excitation and emission slits of 8 and 2 nm, respectively. The CAC value was calculated from the curve obtained by plotting the ratio of the peaks at 373 and 384 nm against the peptide concentrations.

### FTIR spectroscopy

FTIR spectra were collected using a Nicolet Nexus 470 FTIR spectrometer with a DTGS (deuteratedtriglycine sulfate) detector. A 30 μl aliquot of the peptides solution was deposited on a polyethylene IR card and dried under vacuum. Measurements were taken using a 4 cm^−1^ resolution and by averaging 64 scans. The absorbance maxima values were determined using an OMNIC analysis program (Nicolet). The background was subtracted using a control spectrum. To record the FTIR spectra of emulsions, a 30 μl aliquot of the peptides solution was deposited on a KBr IR card and dried under vacuum. The background was subtracted using a control spectrum containing only silicone oil on a KBr IR card.

To record the FTIR spectra in solution, peptides were lyophilized twice in 0.5 M HCl solution. Subsequently, the peptides were dissolved in D_2_O (5–7 mg ml^−1^). Emulsions were prepared in the condition described above in D_2_O. Peptide solutions or emulsions were sandwiched between two 25 × 2 mm CaF_2_ windows separated with a 25 μm polytetrafluoroethylene spacer. Measurements were taken using a 4 cm^−1^ resolution and by averaging 128 scans. The background was subtracted using a control D_2_O spectrum.

### Molecular dynamics simulations

All details regarding the construction of the fibril-like model and molecular dynamics simulations procedure are provided in the Supplementary Note 1.

### X-ray crystal structure analysis and crystal data

Single crystals suitable for X-ray diffraction were grown by slow evaporation of the peptide in ethanol (5%)—phosphate buffer (10 mM, pH 7.4) in room temperature and crystals of diffraction quality were obtained after 2–5 days of sample preparation. Crystals suitable for diffraction were coated with Paratone oil (Hampton Research) and mounted on loops and flash frozen in liquid nitrogen. Diffraction data measurements were done on a Bruker KappaApexII system with MoKα radiation at 100(2) K. Data were collected and processed with Apex2 Suite. The structures were solved by direct methods using SHELXT-2013. The structures were refined by full-matrix least squares against F2 with SHELXL-2013. The crystallographic data are given in Supplementary Table 3 and Supplementary Data set 1.

### Accession codes

The X-ray crystallographic coordinates for structures reported in this study have been deposited at the Cambridge Crystallographic Data Centre (CCDC), under deposition numbers CCDC 1473157. These data can be obtained free of charge from the CCDCvia www.ccdc.cam.ac.uk/data_request/cif.

### Wide-angle X-ray scattering

The SHR-FLLF peptide solutions at a concentration of 15 and 5 mg ml^−1^ were sealed in quartz capillaries with a 1.5 mm diameter. WAXS measurements were performed using an in-house X-ray scattering system, with a GeniX (Xenocs) low divergence Cu Kα radiation source (wavelength of 1.54 Å) and a scatterless slits setup^48^. Two-dimensional scattering data, with a momentum transfer wave vector (*q*) range of 0.07−2.5 Å^−1^ at a sample-to-detector distance of about 160 mm, was collected on a Pilatus 300 K detector (Dectris, Baden-Daettwil, Switzerland) and radially integrated using Matlab (MathWorks, Natick, MA, USA)-based procedures (SAXSi). Calibration was performed using silver behenate. The scattering data of the water at pH 2 was collected as background and used to subtract the solvent and spurious scattering from the WAXS system itself, for example, Kapton vacuum windows and air gaps.

### Data availability

The authors declare that all data supporting the findings of this study are available within the article and its Supplementary Information files, or from the corresponding author upon request. The X-ray crystallographic coordinates for structures that support the findings of this study have been deposited in CCDC, with the accession code 1473157.

## Additional information

**How to cite this article:** Mondal, S. *et al*. A minimal length rigid helical peptide motif allows rational design of modular surfactants. *Nat. Commun.* **8,** 14018 doi: 10.1038/ncomms14018 (2017).

**Publisher's note**: Springer Nature remains neutral with regard to jurisdictional claims in published maps and institutional affiliations.

## Supplementary Material

Supplementary Information Supplementary Figures, Supplementary Tables, Supplementary Note and Supplementary References

Supplementary Data 1 CIF for *SHR-FLLF*

## Figures and Tables

**Table 1:** Different physical parameters of oil-in-water emulsions.

**Emulsifiers**	**Peptide sequences**	**Concentrations (mg** **ml**^**−1**^**)**	**Mean hydrodynamic diameter (nm)**	**Zeta potential (mV)**
SHR-FLLF	H_2_N-Phe-Aib-Leu-Ala-Aib-Leu-Phe-OH	5	228±3	+92±2
SHR-FLLF		10	253±5	+82±2
SHR-FLLF		15	219±2	+88±1
SDS	—	2.8	332±1	—
Tween 20	—	1.2	715±6	—
SHR-FLEFL	H_2_N-Phe-Aib-Leu-Glu-Aib-Leu-Phe-OH	5	231±2	+34±1*
SHR-FLEFL		7.5	258±6	+37±2*
SHR-FLEFL		10	238±3	+33±1*
SHR-FLKFL	H_2_N-Phe-Aib-Leu-Lys-Aib-Leu-Phe-OH	5	320±2	+97±2*
SHR-FLKFL		7.5	252±3	+101±3*

^*^See ‘Emulsion preparation and characterization' in Methods.

**Table 2:** A comparative analysis of the different properties of SHR-FLLF, SHR-FLELF and SHR-FLKLF.

**Emulsifiers**	**Peptide sequences**	**Concentrations (mg** **ml**^**−1**^**)**	**Self-assemble**	**Emulsion stability**	**Helical conformation**
SHR-FLLF	H_2_N-Phe-Aib-Leu-Ala-Aib-Leu-Phe-OH	5	Yes	>1 Week	Yes
		10	Yes	>2 Months	Yes
		15	Yes	>2 Months	Yes
SHR-FLEFL	H_2_N-Phe-Aib-Leu-Glu-Aib-Leu-Phe-OH	5	Yes	>1 Week	Yes
		7.5	Yes	>2 Months	Yes
		10	Yes	>2 Months	Yes
SHR-FLKFL	H_2_N-Phe-Aib-Leu-Lys-Aib-Leu-Phe-OH	5	No	<1 Week	Yes
		7.5	No	<1 Week	Yes

## References

[b1] Bibette J., Calderon F. L. & Poulin P. Emulsions: basic principles. Rep. Prog. Phys. 62, 969–1033 (1999).

[b2] Zarzar L. D. . Dynamically reconfigurable complex emulsions via tunable interfacial tensions. Nature 518, 520–524 (2015). 25719669 10.1038/nature14168 PMC4504698

[b3] Kislukhin A. A. . Paramagnetic fluorinated nanoemulsions for sensitive cellular fluorine-19 magnetic resonance imaging. Nat. Mater. 15, 662–668 (2016). 26974409 10.1038/nmat4585 PMC5053764

[b4] Malaki Nik A., Wright A. J. & Corredig M. Interfacial design of protein-stabilized emulsions for optimal delivery of nutrients. Food Funct. 1, 141–148 (2010). 21776464 10.1039/c0fo00099j

[b5] Hanson J. A. . Nanoscale double emulsions stabilized by single-component block copolypeptides. Nature 455, 85–88 (2008). 18769436 10.1038/nature07197

[b6] Rebello S., Asok A. K., Mundayoor S. & Jisha M. S. Surfactants: toxicity, remediation and green surfactants. Environ. Chem. Lett. 12, 275–287 (2014).

[b7] Dickinson E. Hydrocolloids as emulsifiers and emulsion stabilizers. Food Hydrocoll. 23, 1473–1482 (2009).

[b8] Chassaing B. . Dietary emulsifiers impact the mouse gut microbiota promoting colitis and metabolic syndrome. Nature 519, 92–96 (2015). 25731162 10.1038/nature14232 PMC4910713

[b9] Dickinson E. Colloids in food: ingredients, structure, and stability. Annu. Rev. Food Sci. Technol. 6, 211–233 (2015). 25422877 10.1146/annurev-food-022814-015651

[b10] Lam R. S. H. & Nickerson M. T. Food proteins: a review on their emulsifying properties using a structure–function approach. Food Chem. 141, 975–984 (2013). 23790876 10.1016/j.foodchem.2013.04.038

[b11] Dickinson E. Exploring the frontiers of colloidal behaviour where polymers and particles meet. Food Hydrocoll. 52, 497–509 (2016).

[b12] McClements D. J. Protein-stabilized emulsions. Curr. Opin. Colloid Interface Sci. 9, 305–313 (2004).

[b13] Wang L., Walsh M. T. & Small D. M. Apolipoprotein B is conformationally flexible but anchored at a triolein/water interface: a possible model for lipoprotein surfaces. Proc. Natl Acad. Sci. USA 103, 6871–6876 (2006). 16636271 10.1073/pnas.0602213103 PMC1458986

[b14] Raffaini G., Milani R., Ganazzoli F., Resnati G. & Metrangolo P. Atomistic simulation of hydrophobin HFBII conformation in aqueous and fluorous media and at the water/vacuum interface. J. Mol. Graph. Model. 63, 8–14 (2016). 26606320 10.1016/j.jmgm.2015.11.006

[b15] Tucker I. M. . Adsorption of hydrophobin–protein mixtures at the air–water interface: the impact of ph and electrolyte. Langmuir 31, 10008–10016 (2015). 26287651 10.1021/acs.langmuir.5b02403

[b16] Cox P. W. & Hooley P. Hydrophobins: new prospects for biotechnology. Fungal Biol. Rev. 23, 40–47 (2009).

[b17] Dexter A. F. & Middelberg A. P. J. Peptides as functional surfactants. Ind. Eng. Chem. Res. 47, 6391–6398 (2008).

[b18] Dexter A. F., Malcolm A. S. & Middelberg A. P. J. Reversible active switching of the mechanical properties of a peptide film at a fluid-fluid interface. Nat. Mater. 5, 502–506 (2006). 16715085 10.1038/nmat1653

[b19] Xue Y., He L., Middelberg A. P. J., Mark A. E. & Poger D. Determining the structure of interfacial peptide films: comparing neutron reflectometry and molecular dynamics simulations. Langmuir 30, 10080–10089 (2014). 25093605 10.1021/la501715h

[b20] Bai S. . Stable emulsions formed by self-assembly of interfacial networks of dipeptide derivatives. ACS Nano 8, 7005–7013 (2014). 24896538 10.1021/nn501909j

[b21] Scott G. G., McKnight P. J., Tuttle T. & Ulijn R. V. Tripeptide emulsifiers. Adv. Mater. 28, 1381–1386 (2016). 26639675 10.1002/adma.201504697

[b22] Dexter A. F. Interfacial and emulsifying properties of designed β-strand peptides. Langmuir 26, 17997–18007 (2010). 21058648 10.1021/la103471j

[b23] Saito M., Ogasawara M., Chikuni K. & Shimizu M. Synthesis of a peptide emulsifier with an amphiphilic structure. Biosci. Biotechnol. Biochem. 59, 388–392 (1995). 7766174 10.1271/bbb.59.388

[b24] Schafmeister C. E., Miercke L. J. & Stroud R. M. Structure at 2.5 A of a designed peptide that maintains solubility of membrane proteins. Science 262, 734–738 (1993). 8235592 10.1126/science.8235592

[b25] McGregor C.-L. . Lipopeptide detergents designed for the structural study of membrane proteins. Nat. Biotechnol. 21, 171–176 (2003). 12524549 10.1038/nbt776

[b26] Mondal S. . Formation of functional super-helical assemblies by constrained single heptad repeat. Nat. Commun. 6, 8615 (2015). 26468599 10.1038/ncomms9615 PMC4634320

[b27] Frederix P. W. J. M. . Exploring the sequence space for (tri-)peptide self-assembly to design and discover new hydrogels. Nat. Chem. 7, 30–37 (2015). 25515887 10.1038/nchem.2122

[b28] Gazit E. Molecular self-assembly: searching sequence space. Nat. Chem. 7, 14–15 (2015). 25515880 10.1038/nchem.2140

[b29] Aravinda S., Shamala N. & Balaram P. Aib residues in peptaibiotics and synthetic sequences: analysis of nonhelical conformations. Chem. Biodivers. 5, 1238–1262 (2008). 18649312 10.1002/cbdv.200890112

[b30] Tang C., Smith A. M., Collins R. F., Ulijn R. V. & Saiani A. Fmoc-diphenylalanine self-assembly mechanism induces apparent pKa shifts. Langmuir 25, 9447–9453 (2009). 19537819 10.1021/la900653q

[b31] Shrestha R. G. . Peptide-based gemini amphiphiles: phase behavior and rheology of wormlike micelles. Langmuir 28, 15472–15481 (2012). 23075203 10.1021/la3022358

[b32] Kong J. & Yu S. Fourier transform infrared spectroscopic analysis of protein secondary structures. Acta Biochim. Biophys. Sin. (Shanghai) 39, 549–559 (2007). 17687489 10.1111/j.1745-7270.2007.00320.x

[b33] Kennedy D. F., Crisma M., Toniolo C. & Chapman D. Studies of peptides forming 3_10_-helices and α-helices and β-bend ribbon structures in organic solution and in model biomembranes by fourier-transform infrared-spectroscopy. Biochemistry 30, 6541–6548 (1991). 2054352 10.1021/bi00240a026

[b34] Zhang S. Lipid-like self-assembling peptides. Acc. Chem. Res. 45, 2142–2150 (2012). 22720818 10.1021/ar300034v

[b35] Vauthey S., Santoso S., Gong H., Watson N. & Zhang S. Molecular self-assembly of surfactant-like peptides to form nanotubes and nanovesicles. Proc. Natl Acad. Sci. USA 99, 5355–5360 (2002). 11929973 10.1073/pnas.072089599 PMC122773

[b36] Zhang S. Fabrication of novel biomaterials through molecular self-assembly. Nat. Biotechnol. 21, 1171–1178 (2003). 14520402 10.1038/nbt874

[b37] Fatouros D. G. . Lipid-like self-assembling peptide nanovesicles for drug delivery. ACS Appl. Mater. Interfaces 6, 8184–8189 (2014). 24821330 10.1021/am501673x PMC4059226

[b38] Tantakitti F. . Energy landscapes and functions of supramolecular systems. Nat. Mater. 15, 469–476 (2016). 26779883 10.1038/nmat4538 PMC4805452

[b39] Ortony J. H. . Internal dynamics of a supramolecular nanofibre. Nat. Mater. 13, 812–816 (2014). 24859643 10.1038/nmat3979 PMC4110180

[b40] Moyer T. J. . pH and amphiphilic structure direct supramolecular behavior in biofunctional assemblies. J. Am. Chem. Soc. 136, 14746–14752 (2014). 25310840 10.1021/ja5042429 PMC4210119

[b41] Hartgerink J. D., Beniash E. & Stupp S. I. Self-assembly and mineralization of peptide-amphiphile nanofibers. Science 294, 1684–1688 (2001). 11721046 10.1126/science.1063187

[b42] Kallio J. M., Linder M. B. & Rouvinen J. Crystal structures of hydrophobin HFBII in the presence of detergent implicate the formation of fibrils and monolayer films. J. Biol. Chem. 282, 28733–28739 (2007). 17636262 10.1074/jbc.M704238200

[b43] Kawaguchi M. Silicone oil emulsions stabilized by polymers and solid particles. Adv. Colloid Interface Sci. 233, 186–199 (2016). 26170165 10.1016/j.cis.2015.06.005

[b44] Paximada P., Koutinas A. A., Scholten E. & Mandala I. G. Effect of bacterial cellulose addition on physical properties of WPI emulsions. comparison with common thickeners. Food Hydrocoll. 54, 245–254 (2016).

[b45] Philipse A. P. The random contact equation and its implications for (colloidal) rods in packings, suspensions, and anisotropic powders. Langmuir 12, 1127–1133 (1996).

[b46] Dickinson E. & Golding M. Rheology of sodium caseinate stabilized oil-in-water emulsions. J. Colloid Interface Sci. 191, 166–176 (1997). 9241217 10.1006/jcis.1997.4939

[b47] Dominguez A., Fernandez A., Gonzalez N., Iglesias E. & Montenegro L. Determination of critical micelle concentration of some surfactants by three techniques. J. Chem. Educ. 74, 1227–1231 (1997).

[b48] Li Y., Beck R., Huang T., Choi M. C. & Divinagracia M. Scatterless hybrid metal-single-crystal slit for small-angle X-ray scattering and high-resolution X-ray diffraction. J. Appl. Crystallogr. 41, 1134–1139 (2008).

