# Supplementary material for: A minimal length rigid helical peptide motif allows rational design of modular surfactants
Source: Nat Commun. 2017 Jan 13;8:14018. doi: 10.1038/ncomms14018 (PMC5241864; doi:10.1038/ncomms14018)
Supplement: Supplementary Information — Supplementary Figures, Supplementary Tables, Supplementary Note and Supplementary References [file ncomms14018-s1.pdf]

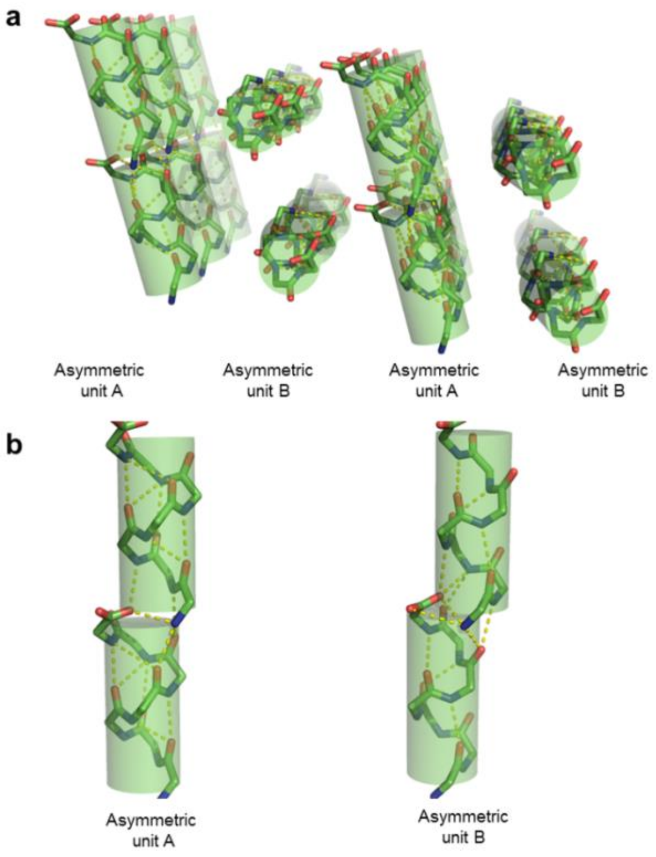

Supplementary Figure 1. **Detailed crystal packing pattern of SHR-FLLF**. (a) Asymmetric units A and B packed in end-to-end fashion to reveal a continuous column with helical backbone. Two such adjacent helical columns interacted only through hydrophobic and stacking interactions and did not involve any H-bonding interactions. (b) A discrete head-to-tail H-bonding pattern observed in asymmetric unit A and asymmetric unit B, respectively.

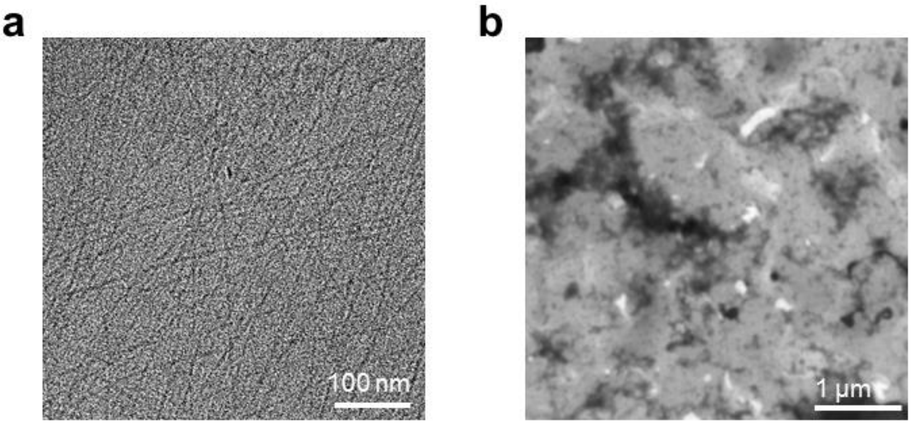

Supplementary Figure 2. **Transmission Electron micrograph of SHR-FLLF in different pH.** (a) Cryo-TEM image of SHR-FLLF at 20 $mg.ml^{-1}$ at pH 1.5 in aqueous solution. (b) TEM image of SHR-FLLF (5 $mg.ml^{-1}$) in ethanol (5%) - phosphate buffer (10 mM, pH 7.4)

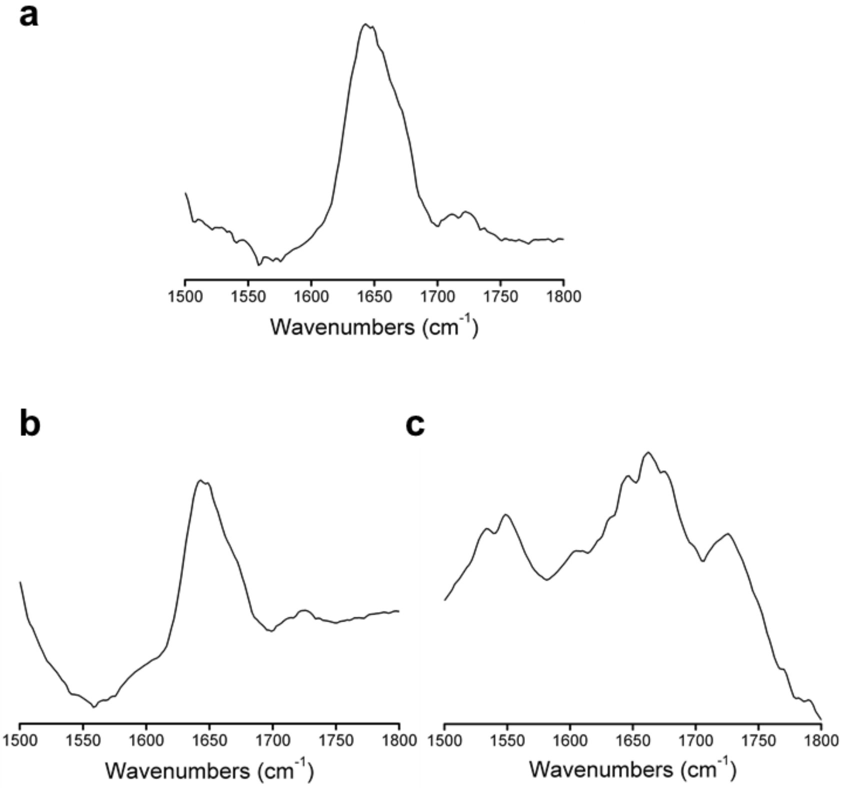

Supplementary Figure 3. FTIR spectra of SHR-FLLF in different conditions. (a) SHR-FLLF peptide was dissolved in D_2O and spectrum was recorded in solution state. (b) Emulsion was prepared in D_2O and spectrum was taken in solution state. (c) Emulsion was prepared in H_2O and spectrum was taken in dry condition.

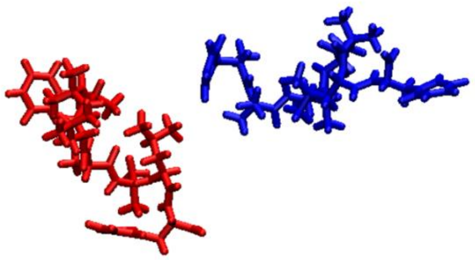

Supplementary Figure 4. Inter-fibrils interactions observed in molecular dynamic simulation of SHR-FLLF. A zoom in of hydrophobic interactions viewed between the first layer and the second layer**.**

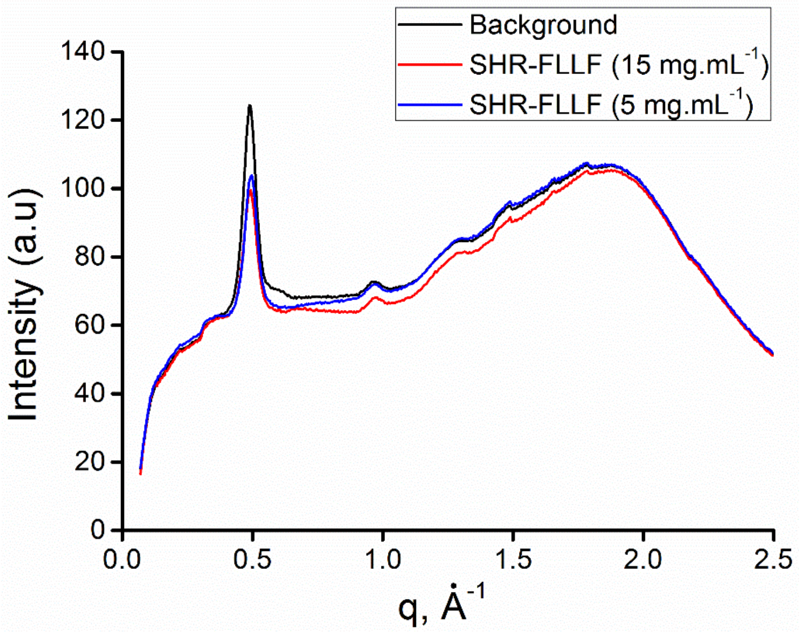

Supplementary Figure 5. WAXS signals of SHR-FLLF at two different concentrations revealed similar scattering pattern as background solvent. The peak seen at approximately q = 0.5 $Å^{-1}$ pertains to the Kapton vacuum windows.

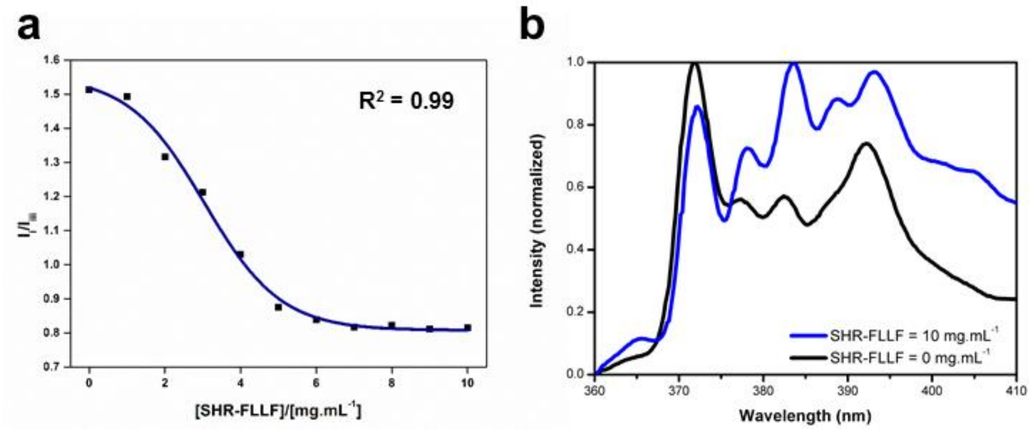

Supplementary Figure 6. Determination of critical aggregation concentration (CAC) of SHR-FLLF. (a) Plot of ratio of vibrational band intensities (I_i/I_{iii}) as a function of [SHR-FLLF]. (b) Fluorescence emission spectrum of pyrene in presence and absence of SHR-FLLF. Pronounced increase in third vibrational band intensity was observed in presence of peptide.

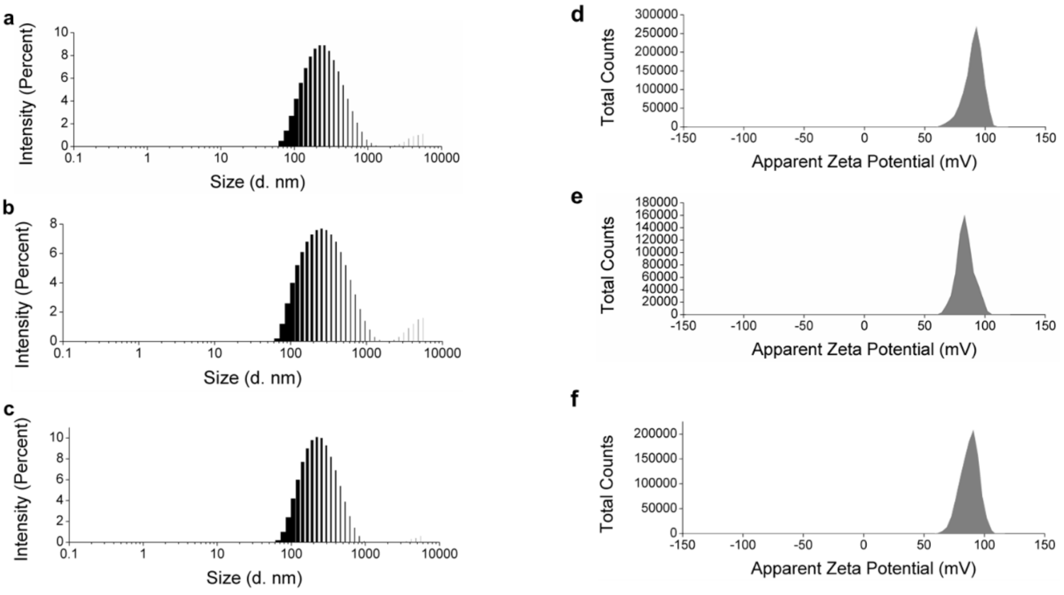

Supplementary Figure 7. Droplet size distribution and zeta potential of emulsions prepared with SHR-FLLF at different concentrations. (a-c) Distribution of hydrodynamic diameter of emulsions at 5 $mg.ml^{-1}$, 10 $mg.ml^{-1}$ and 15 $mg.ml^{-1}$ of [SHR-FLLF], respectively (top to bottom). (d-f) Apparent zeta potential of emulsions at 5 $mg.ml^{-1}$, 10 $mg.ml^{-1}$ and 15 $mg.ml^{-1}$ of [SHR-FLLF], respectively (top to bottom).

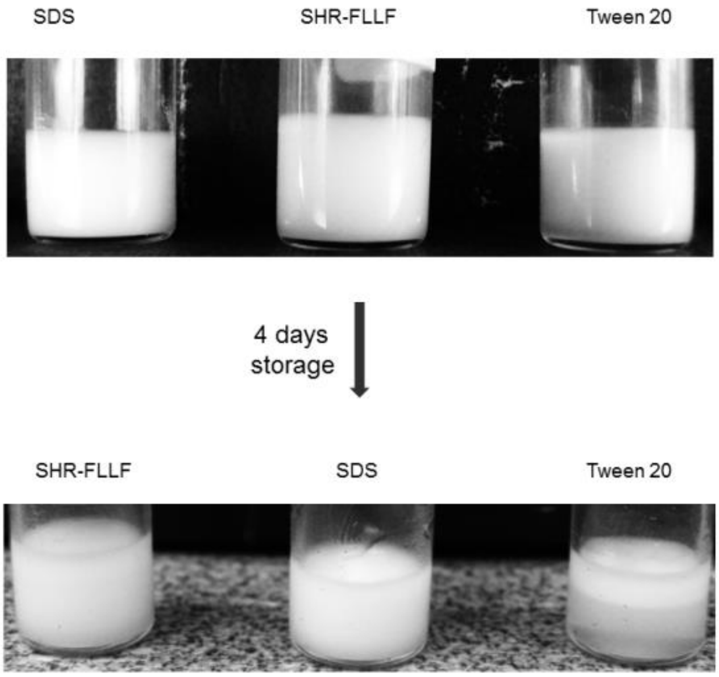

Supplementary Figure 8. Comparative stability assessment of silicone oil (20 %)-water (80 %) emulsions prepared with SDS (2.8 mg.mL^{-1}), SHR-FLLF (5.0 mg.mL^{-1}) and tween 20 (1.2 mg.mL^{-1}). (Top) photographic images of freshly prepared emulsions and (bottom) photographic images of emulsions stored for 4 days at 25 °C.

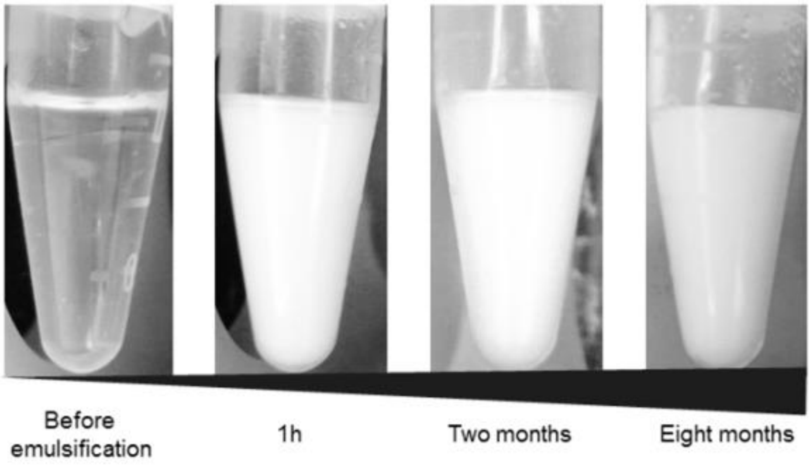

Supplementary Figure 9. Photographic images showing the long-term stability of SHR-FLLF (10 mg.ml^{-1}) **stabilized emulsion**.

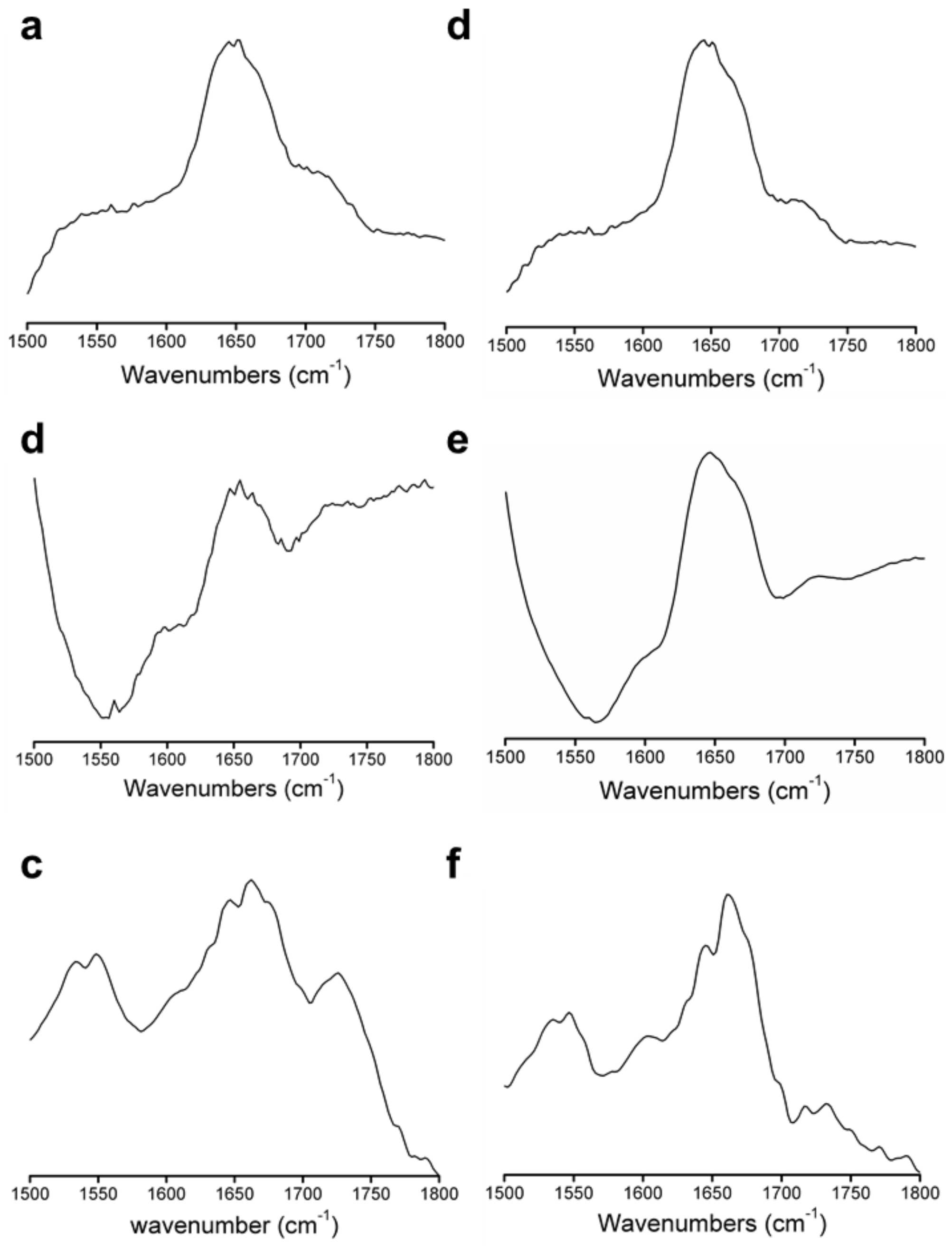

Supplementary Figure 10. FTIR spectra of SHR-FLELF and SHR-FLKLF in different conditions. (a-c) Studies with SHR-FLELF sequence. (a) SHR-FLELF was dissolved in D_2O and spectrum was recorded in solution state. (b) Emulsion was prepared in D_2O and spectrum was taken in solution state. (c) Emulsion was prepared in H_2O and spectrum was taken in dry condition. (d-f) Studies with SHR-FLKLF sequence. (d) SHR-FLKLF was dissolved in D_2O and spectrum was recorded in solution state. (e) Emulsion was prepared in D_2O and spectrum was taken in solution state. (f) Emulsion was prepared in H_2O and spectrum was taken in dry condition.

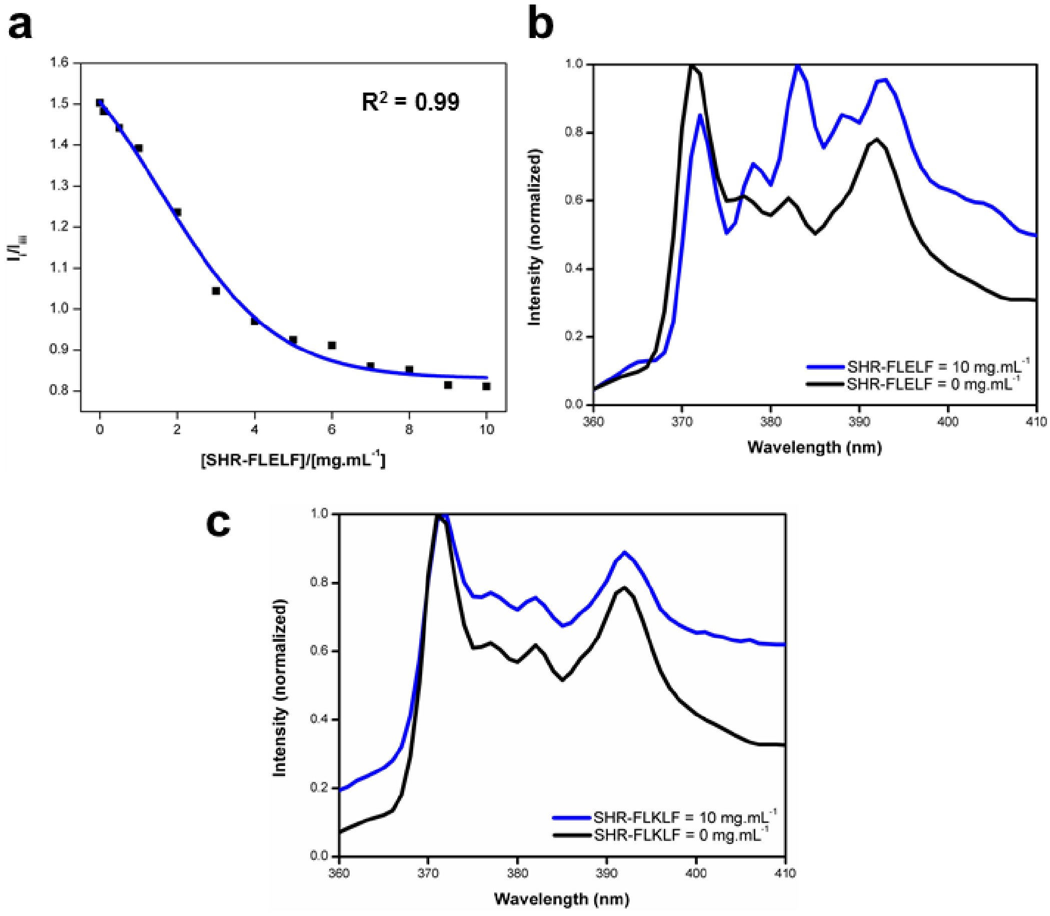

Supplementary Figure 11. Determination of critical aggregation concentration (CAC) and self-assembly characteristic of SHR-FLELF and SHR-FLKLF. (a) Plot of ratio of vibrational band intensities (I_i/I_{iii}) as a function of [SHR-FLELF]. (b) Fluorescence emission spectrum of pyrene in presence and absence of SHR-FLELF showed pronounced increased in third vibrational band intensity in presence of peptide. (c) Fluorescence emission spectrum of pyrene in presence and absence of SHR-FLKLF showed that the peptide has very minor effect on vibrational ban intensities and primarily exists in non-aggregation state.

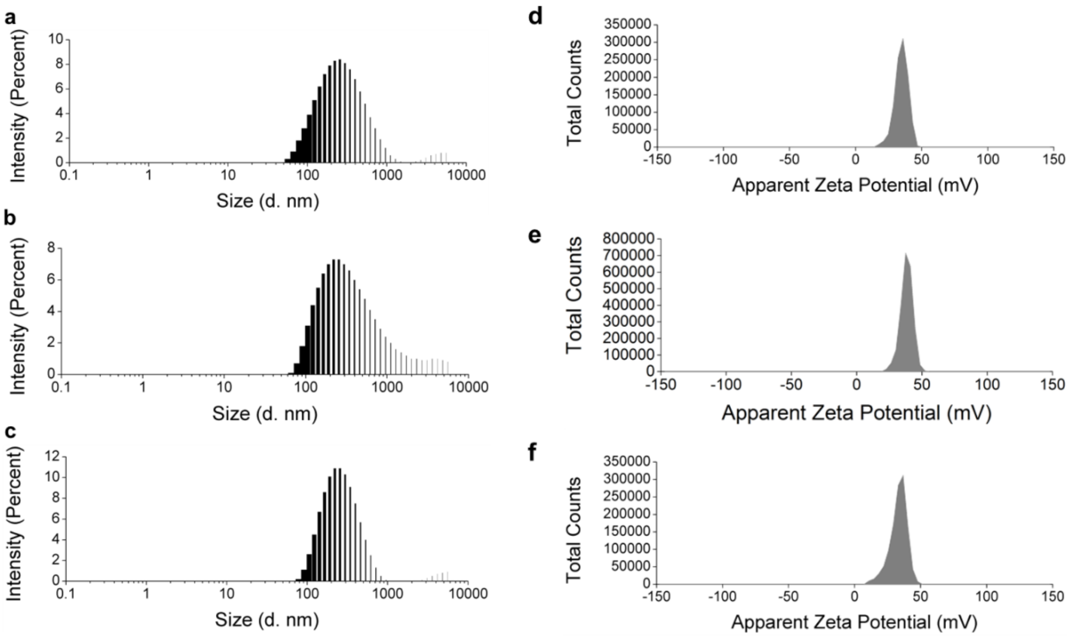

Supplementary Figure 12. Droplet size distribution and zeta potential of emulsions prepared with SHR-FLELF at different concentrations. (a-c) Distribution of hydrodynamic diameter of emulsions at 5 mg.ml^{-1}, 7.5 mg.ml^{-1} and 10 mg.ml^{-1} of [SHR-FLELF], respectively (top to bottom). (d-f) Apparent zeta potential of emulsions at 5 mg.ml^{-1}, 7.5 mg.ml^{-1} and 10 mg.ml^{-1} of [SHR-FLELF], respectively (top to bottom).

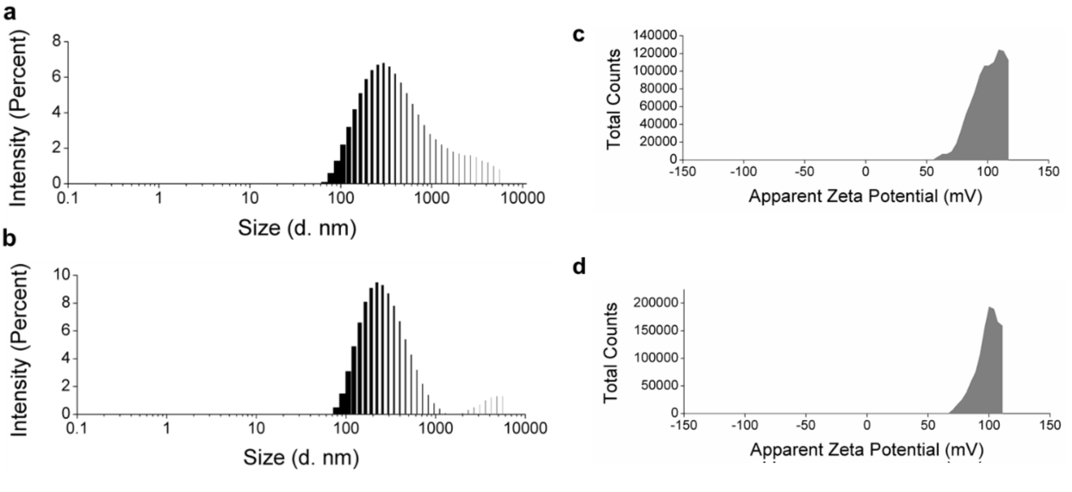

Supplementary Figure 13. Droplet size distribution and zeta potential of emulsions prepared with SHR-FLKLF at different concentrations. (a-b) Distribution of hydrodynamic diameter of emulsions at 5 mg.ml^{-1} and 7.5 mg.ml^{-1} of [SHR-FLKLF], respectively (top to bottom). (d-f) Apparent zeta potential of emulsions at 5 mg.ml^{-1} and 7.5 mg.ml^{-1} of [SHR-FLKLF], respectively (top to bottom).

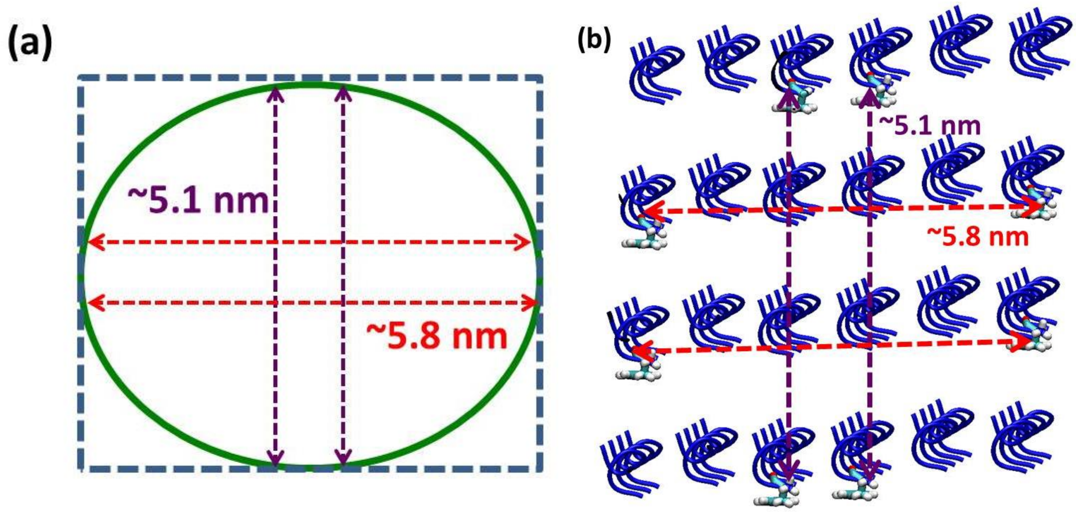

Supplementary Figure 14. (a) A scheme of the procedure to construct the fibril-like self-assemble structural model (b) The initial fibril-like self-assemble structural model. The peptides are seen in blue. Each layer consists of 4 peptides by 6 peptides. A total of four layers are seen. The diameter values were measured from Cα of Phe1 to Cα of Phe1 from the ends of the structural model in two 'centers' of the fibril-like structure.

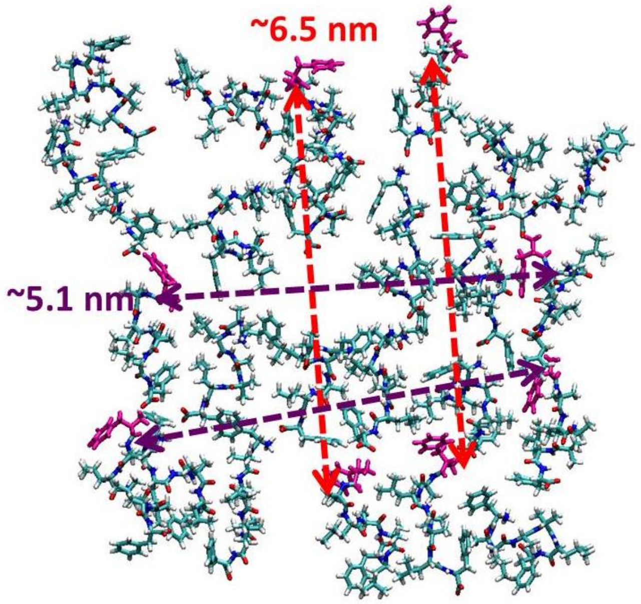

Supplementary Figure 15. This image shows the final simulated fibril-like structure. The diameter values were measured from Cα of Phe1 to Cα of Phe1 from the ends of the structural model in two 'centers' of the fibril-like structure.

Supplementary Table 1. Detailed torsion angles of two asymmetric molecules present in the crystal of SHR-FLLF. Values were reported starting from C-terminus Leu to N-terminus Aib.

	SHR-FLLF			
Amino acid residues	Asymmetric unit A		Asymmetric unit B	
	φ	ψ	φ	ψ
**Leu**	**-87.2**	**-24.5**	**-85.8**	**-2.8**
**Aib**	**-52.6**	**-44.7**	**-54.1**	**-29.7**
**Ala**	**-56.2**	**-43.5**	**-54.1**	**-39.0**
**Leu**	**-69.8**	**-42.3**	**-81.6**	**-42.2**
**Aib**	**-52.2**	**-43.6**	**-53.5**	**-46.1**
Average	-63.6	-39.7	-65.8	-31.9

Supplementary Table 2. Comparison of the characteristic of peptide zeta potentials at different pH.

**Emulsifiers**	**Peptide Sequences**	**Concentrations**	**pH**	
			**Zeta potential (mV)**	
SHR-FLLF	H_2N-Phe-Aib-Leu-Ala-Aib-Leu-Phe-OH	5 $mg.mL^{-1}$	2.77	1.74
			+37 ± 1	+33 ± 1
SHR-FLEFL	H_2N-Phe-Aib-Leu-Glu-Aib-Leu-Phe-OH	5 $mg.mL^{-1}$	2.61	1.85
			+47 ± 3	+42 ± 6
SHR-FLKFL	H_2N-Phe-Aib-Leu-Lys-Aib-Leu-Phe-OH	5 $mg.mL^{-1}$	2.71	1.72
			+21 ± 3	+15 ± 2

Supplementary Table 3. Crystal data and structure refinement for SHR-FLLF.

Identification code	shimon34cif
Empirical formula	C82 H145 N14 O26.50
Formula weight	1751.11
Temperature	100(2) K
Wavelength	0.71073 A
Crystal system, space group	Triclinic, P1
Unit cell dimensions	a = 11.0081(10) A alpha = 81.370(6) deg.<br>b = 11.0439(11) A beta = 78.531(5) deg.<br>c = 20.174(2) A gamma = 80.367(6) deg.
Volume	2352.3(4) A^3
Z, Calculated density	1, 1.236 Mg/m^3
Absorption coefficient	0.092 mm^-1
F(000)	947
Crystal size	0.150 x 0.020 x 0.020 mm
Theta range for data collection	2.865 to 25.680 deg.
Limiting indices	-13<=h<=11, -13<=k<=13, -24<=l<=24
Reflections collected / unique	28139 / 15363 [R(int) = 0.0396]
Completeness to theta = 25.242	98.2 %
Absorption correction	Semi-empirical from equivalents
Max. and min. transmission	0.9982 and 0.9864
Refinement method	Full-matrix least-squares on F^2
Data / restraints / parameters	15363 / 3 / 1147
Goodness-of-fit on F^2	1.028
Final R indices [I>2sigma(I)]	R1 = 0.0556, wR2 = 0.1123
R indices (all data)	R1 = 0.0826, wR2 = 0.1266
Extinction coefficient	n/a
Largest diff. peak and hole	0.594 and -0.567 e.A^-3

Supplementary note 1

Computation Details

Construction of the fibril-like model

One of the most challenging issues when investigating the self-assembly of peptides at the atomic resolution is to predict the 'building block' structural model of the monomeric peptide. In the current study, the 'building block' structural model was taken from the crystal structure. The fibril-like structural model was constructed using the Accelrys Discovery Studio software (http://accelrys.com/products/discovery-studio/).

The initial model was constructed by considering the diameter dimensions that have been observed by the Cryo-TEM measurement; therefore our constructed fibril-like structural model was originally based initially from the cryo-TEM results. The fibril-like structural model was constructed by forming four layers, in which each layer consists of 4 peptides by 6 peptides (i.e. a total of 24 peptides) in order to form a diameter of around 5-6 nm (as observed by the Cryo-TEM measurements). Therefore, the fibril-like structural model consists of 96 peptides. Each peptide consists of 7 amino acids; thus, the total system consists of 672 amino acids. We therefore constructed the fibril-like structural model with diameter values of 5.8 nm (the 6 peptides) by 5.1 nm (the 4 peptides). The diameter values were estimated from the ends of the fibril model of 4 by 6, considering the distances between Cα atom of Phe1 (Supplementary Fig. 14). The four peptides in each layer form π-π interactions between the two Phe groups. It should be noted here, that while modeling the fibril-like structure we consider proximity between the Phe groups, as suggested by experiment. The six peptides in each layer form hydrophobic interactions between the Aib residue and the Ala residue. After simulations, the diameter values of the fibril-like structure are 6.2 nm by 5.1 nm – indicating that the diameter values were conserved along the simulations.

Molecular dynamics (MD) simulations procedure

All-atom explicit MD simulations of the solvated model were performed in NPT ensemble using the NAMD program[1] with the CHARMM36 force-field[2–4] with CMAP corrections for 20 ns. The parameters for the Aib residues were obtained from the MacKerell lab. We integrated these parameters to the force-field potential. The model were explicitly solvated with TIP3P water molecules[5,6]. The Langevin piston method [1,7,8] with a decay period of 100 fs, and a damping time of 50 fs was used to maintain a constant pressure of 1 atm. The temperature (310 K) was controlled by Langevin thermostat with a damping coefficient of 10 ps^{-1}[1]. The short-range van der Waals (VDW) interactions were calculated using the switching function, with a twin range cutoff of 10.0 and 12.0 Å. Long-range electrostatic interactions were calculated using the particle mesh Ewald method with a cut-off of 12.0 Å for all simulations[9,10]. The equations of motion were integrated using the leapfrog integrator with a step of 1 fs. All initial variant model was energy minimized and then solvated in a TIP3P water box with a minimum distance of 15 Å from any edge of the box to any peptide atom. Any water molecule within 2.5 Å of the systems was removed. Counterions were not added at random locations, since the systems already are neutralized with no charge.

The solvated systems were energy minimized for 2000 conjugated gradient steps. The hydrogen atoms were constrained to the equilibrium bond using the SHAKE algorithm[11]. The minimized solvated systems were heated at 150 K, where all atoms were allowed to move. Then, the systems were heated from 150 K to 250 K for 300 ps and equilibrated at 310 K for 300 ps. These conditions (20 ns and 310K) were applied to the examined fibril-like structure and illustrated no change in the simulations, thus provided a stable fibril-like structure. As seen in supplementary figure 15, the initial diameter values (shown in Supplementary Fig. 14) have not change during the simulations (Supplementary Fig.

15), therefore justifying the timescale of the simulations. The final diameter values after simulations are 6.5 and 5.1 nm, which are similar to the observed values that have been measure by the Cryo-TEM. This indicates that the diameter values were conserved within the timescale of the simulations.

Supplementary References

1. Kalé, L. *et al.* NAMD2: Greater scalability for parallel molecular dynamics. *J. Comput. Phys.* **151,** 283–312 (1999).

2. Best, R. B., Buchete, N.-V. & Hummer, G. Are current molecular dynamics force fields too helical? *Biophys. J.* **95,** L07–L09 (2008).

3. Mackerell, A. D. *et al.* All-atom empirical potential for molecular modeling and dynamics studies of proteins. *J. Phys. Chem. B* **102,** 3586–3616 (1998).

4. MacKerell, A. D., Feig, M. & Brooks, C. L. Improved treatment of the protein backbone in empirical force fields. *J. Am. Chem. Soc.* **126,** 698–699 (2004).

5. Jorgensen, W. L., Chandrasekhar, J., Madura, J. D., Impey, R. W. & Klein, M. L. Comparison of simple potential functions for simulating liquid water. *J. Chem. Phys.* **79,** 926–935 (1983).

6. Mahoney, M. W. & Jorgensen, W. L. A five-site model for liquid water and the reproduction of the density anomaly by rigid, nonpolarizable potential functions. *J. Chem. Phys.* **112,** 8910–8922 (2000).

7. Martyna, G. J., Tobias, D. J. & Klein, M. L. Constant pressure molecular dynamics algorithms. *J. Chem. Phys.* **101,** 4177–4189 (1994).

8. Feller, S. E., Zhang, Y., Pastor, R. W. & Brooks, B. R. Constant pressure molecular dynamics simulation: The Langevin piston method. *J. Chem. Phys.* **103,** 4613–4621 (1995).

9. Darden, T., York, D. & Pedersen, L. Particle mesh Ewald: An N·log(N) method for Ewald sums in large systems. *J. Chem. Phys.* **98,** 10089–10092 (1993).

10. Essmann, U. *et al.* A smooth particle mesh Ewald method. *J. Chem. Phys.* **103,** 8577–8593 (1995).

11. Ryckaert, J. P., Ciccotti, G. & Berendsen, H. J. C. Numerical-integration of cartesian equations of motion of a system with constraints-molecular-dynamics of n-alkanes. *J. Comput. Phys.* **23,** 327–341 (1977).
